# Role and Function of Peroxisomes in Neuroinflammation

**DOI:** 10.3390/cells13191655

**Published:** 2024-10-05

**Authors:** Chinmoy Sarkar, Marta M. Lipinski

**Affiliations:** 1 Shock, Trauma and Anesthesiology Research (STAR) Center, Department of Anesthesiology, University of Maryland School of Medicine, Baltimore, MD 21201, USA; 2 Shock, Trauma and Anesthesiology Research (STAR) Center, Department of Anesthesiology and Department of Anatomy and Neurobiology, University of Maryland School of Medicine, Baltimore, MD 21201, USA; mlipinski@som.umaryland.edu

**Keywords:** peroxisomes, neuroinflammation, peroxisomal disorders, very-long-chain fatty acids, ether phospholipids

## Abstract

Peroxisomes are organelles involved in many cellular metabolic functions, including the degradation of very-long-chain fatty acids (VLCFAs; C ≥ 22), the initiation of ether-phospholipid synthesis, and the metabolism of reactive oxygen species. All of these processes are essential for the maintenance of cellular lipid and redox homeostasis, and their perturbation can trigger inflammatory response in immune cells, including in the central nervous system (CNS) resident microglia and astrocytes. Consistently, peroxisomal disorders, a group of congenital diseases caused by a block in peroxisomal biogenesis or the impairment of one of the peroxisomal enzymes, are associated with neuroinflammation. Peroxisomal function is also dysregulated in many neurodegenerative diseases and during brain aging, both of which are associated with neuroinflammation. This suggests that deciphering the role of peroxisomes in neuroinflammation may be important for understanding both congenital and age-related brain dysfunction. In this review, we discuss the current advances in understanding the role and function of peroxisomes in neuroinflammation.

## 1. Introduction

Peroxisomes are single-membrane-bound organelles that play a crucial role in maintaining cellular lipid metabolism and redox balance [1,2,3]. They were first identified by Rhodin et al. as micro-bodies in an electron microscopic study in 1954 [3,4]. Later, Christian de Duve, who received the Nobel Prize for his discovery of lysosomes, named them as peroxisomes when he and his colleagues detected the presence of peroxide-generating and -degrading enzymes in a peroxisome-enriched fraction isolated from a rat liver [4]. Initially, peroxisomes were considered redundant organelles that were needed for fatty acid degradation when mitochondrial function was disrupted or during fatty acid overload that overwhelmed mitochondrial fatty acid oxidation [3]. Their importance was discovered following the identification of peroxisomal disorders, a group of congenital diseases caused by deficiencies in peroxisomal proteins and associated with severe pathological phenotypes including neurological impairment, neurodegeneration, and neuroinflammation [2,3].

**Figure 1:**
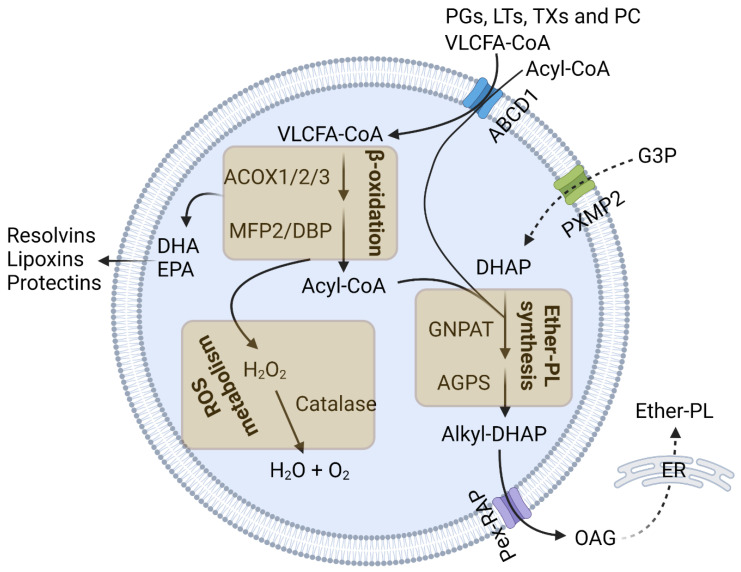
Peroxisomal functions. Peroxisomes degrade very-long-chain fatty acids (VLCFAs; C ≥ 22) by β-oxidation using acyl-CoA oxidase 1/2/3 (ACOX1/2/3) and multifunctional protein 2 (MFP2)/D-bifunctional protein (DBP). Peroxisomal β-oxidation also degrades the following inflammatory lipid metabolites: prostaglandins (PGs), leukotrienes (LTs), thromboxanes (TXs), and prostacyclin (PC). It is also required for the synthesis of docosahexaenoic acid (DHA) and eicosapentaenoic acid (EPA). Peroxisomes initiate ether-PL synthesis. The peroxisomal enzymes glyceronephosphate O-acyltransferase (GNPAT) and alkylglycerone phosphate synthase (AGPS) synthesize the ether-PL precursor 1-O-alkyl glycerol (OAG) from dihydroxyacetone phosphate (DHAP). Peroxisomes also maintain the cellular ROS level. The peroxisomal enzyme catalase decomposes hydrogen peroxides. This figure was created with Biorender.com.

**Figure 2:**
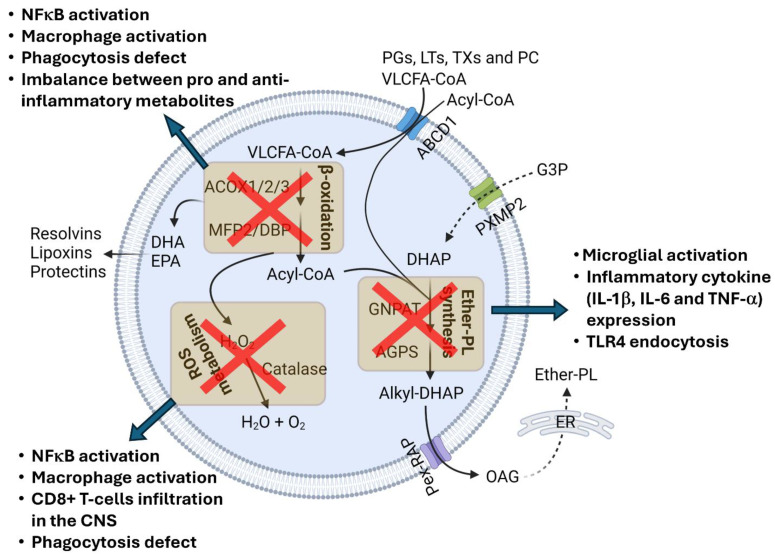
Peroxisomal impairment triggers inflammatory responses. An impairment to peroxisomal β-oxidation and ROS metabolism causes macrophage activation, the stimulation of the NFκB pathway, and phagocytosis defects. CD8+ T-cell infiltration in the CNS is associated with impaired peroxisomal ROS metabolism. Disruption in peroxisomal ether-PL synthesis activates the microglia, inducing inflammatory cytokine expression and TLR4 endocytosis. This figure was created with Biorender.com.

Inflammation of the central nervous system (CNS), or neuroinflammation, arises in response to neuronal injury, infection, stress, or disease [5,6,7,8]. It triggers the proliferation and activation of CNS-resident microglia and astrocytes and may include the migration of peripheral macrophages to the CNS [5,6,7,8]. Inflammatory response is regulated by many factors, including oxidative stress and several types of lipid metabolites. Peroxisomes play an important role in maintaining cellular redox balance and lipid metabolism [1,2,3] (Figure 1). They are essential to the degradation of very-long-chain fatty acids (VLCFAs; C ≥ 22) and branched-chain fatty acids by β- and α-oxidation, respectively [1,2,3,9]. They also initiate the biosynthesis of ether-phospholipids (ether-PLs), an ether-bond containing glycerophospholipids that are major components of cellular membrane lipid rafts [1,2,3,9,10]. Peroxisomal enzyme catalase decomposes hydrogen peroxide and restricts cellular oxidative stress [1,2,3]. All these functions play important roles in regulating the inflammatory response of immune cells, and their perturbation can trigger inflammation (Figure 2). In this review, we discuss the role and functions of peroxisomes in neuroinflammation.

## 2. Peroxisomal Functions in Immune Response

### 2.1. Peroxisomal β-Oxidation

Peroxisomal β-oxidation is essential to VLCFA degradation. VLCFAs are transported to peroxisomes as CoA esters through peroxisomal membrane resident transporter ATP-binding cassette transporter subfamily D member 1 (ABCD1) [1,2,3,11,12]. They undergo chain shortening inside the peroxisomal lumen by acyl-CoA oxidase 1 (ACOX1)- and multifunctional protein 2 (MFP2)-mediated β-oxidation, followed by transport to the mitochondria for the complete degradation to CO2 and water [1,2,9,12] (Figure 1). The perturbation of peroxisomal β-oxidation causes the accumulation of VLCFAs [1,2]. Elevated levels of VLCFAs may affect phagocytic processes in immune cells by interfering with membrane reorganization [13]. Recently, Nath et al. demonstrated that peroxisomal impairment, particularly the depletion of the peroxisomal β-oxidation enzyme thiolase alters the membrane lipid composition and prevents membrane protrusion during infection affecting phagocytosis [14]. They also showed that peroxisomal depletion impairs the Rho1 signaling required for cytoskeleton organization for phagocytosis and cytokine secretion. Since phagocytosis by immune cells clear pathogenic protein aggregates, damaged tissue debris, and pathogenic organisms, impairment to the phagocytic process due to VLCFA accumulation triggers an inflammatory response (Figure 2).

Multiple studies have demonstrated the VFCLA-mediated activation of inflammatory signaling (Figure 2). Treatment with saturated VLCFAs triggers an inflammatory response in macrophages by activating c-Jun Kinase [15]. A block in peroxisomal function, including the decreased β-oxidation of VLCFAs by the knockdown of the peroxisomal enzyme of β-oxidation pathway, multifunctional protein 2 (MFP2), has been shown to potentiate a Lipopolysaccharide (LPS)-induced proinflammatory response in macrophages [16]. Chung and Ye et al. recently demonstrated that VLCFA accumulation in glia similarly triggers neuroinflammation in a fly model [17]. They showed that a loss of function mutation in *Acox1* that encodes for the peroxisomal enzyme for β-oxidation, acyl-coA oxidase 1 (ACOX1), increased VLCFAs in glia, causing an increase in the glial spingosine-1-phosphate (S1P) level. This caused NF-κB activation and induced the infiltration by peripheral macrophages of the brain [17]. On the other hand, proinflammatory stimuli can also affect VLCFA levels. LPS treatment in macrophages has also been linked to elevated levels of saturated or mono-saturated VLCFAs due to a lowered peroxisomal β-oxidation rate.

In addition to the catabolic function in degrading VLCFAs, peroxisomal β-oxidation is also involved in the synthesis of bioactive lipid metabolites including docosahexaenoic acid (DHA) or eicosapentaenoic acid (EPA) [13], which have immunosuppressive and anti-inflammatory properties. DHA and EPA bind to the G-protein-coupled receptors of different immune cells including macrophages, monocytes, neurotrophils, B-lymphocytes, T-lymphocytes, and NKT cells and modulate their immune response [13]. They also activate the nuclear receptor PPARγ (peroxisome proliferator activate receptor gamma) and RXR (retinoid X receptor), which play a crucial role in resolving inflammatory response [13,18]. They have also been shown to restrict NF-κB activation in RAW 264.7 macrophage cell lines [13]. Both DHA and EPA are also precursors of different anti-inflammatory and immunomodulatory lipid metabolites. DHA forms anti-inflammatory resolvins (D1–D4), protectins, maresin, lipoxin, and elovanoid, while EPA forms anti-inflammatory resolvins and proinflammatory prostaglandins (PGs), leukotrienes (LTs), thromboxanes (TXs), and prostacyclin (PC). These immunomodulatory lipid metabolites are also degraded by peroxisomal β-oxidation [13], the perturbation of which disrupts the balance between pro- and anti-inflammatory metabolites and may trigger inflammatory response (Figure 2).

### 2.2. Ether-PL Synthesis

Ether-PLs are unique glycerophospholipids that contain an ether-bond at the sn-1 position of their glycerol backbone [10,19]. Based on the presence of unsaturation next to the ether-bond, ether-PLs are classified into two types: alkyl and alkenyl/vinyl ether-PLs [10,19]. In alkenyl- or vinyl-ether-PLs, also called plasmalogens, a cis double bond is present next to the ether bond, while alkyl ether-PLs do not contain unsaturation next to the ether bond [10,19]. Ether-PLs are major constituent of the membrane lipid raft; they regulate cellular signaling pathways and serve as antioxidants [10,19]. The initial steps of ether-PL synthesis are carried out within peroxisomes [1,3,9,10,19]. Peroxisomal luminal enzyme–glyceronephosphate synthase (GNPAT) catalyzes the acylation of dihydroxyacetone phosphate (DHAP) at the sn-1 position using long-chain fatty acyl CoAs to generate acyl-DHAP [1,10,13,19]. In the next step, alkylgycerone phosphate synthase (AGPS) replaces this acyl group with an alkyl group to generate an ether bond containing 1-O-alkyl-DHAP (Figure 1). Alkyl groups are provided by the fatty alcohols generated by the peroxisomal membrane associated fatty-acid-reducing enzymes, fatty acyl CoA reductase 1 and 2 (FAR1/2). The final peroxisomal step of ether-PL synthesis is carried out by acyl/alkyl dihydroxycetone phosphate reductase, which reduces 1-O-alkyl-DHAP to 1-O-alkyl-glycerol (OAG). OAG is transported to the ER, where the remaining steps of ether-PL synthesis are completed [1,10,13,19].

Ether-PLs are highly abundant in the brain, constituting almost 20% of brain phospholipids [10]. They are major components of the myelin structure [10,19]. They form a strong hydrogen bond due to the absence of carbonyl oxygen at the sn-1 position. The vinyl ether bond in plasmalogens facilitates the close alignment of sn-1 and sn-2 chains. This provides structural rigidity and compactness in the myelin structure. The perturbation of ether-PL homeostasis disrupts neuronal function and triggers neuroinflammation [10,19] (Figure 2). Plasmalogens, the vinyl-ether-bond-containing ether-PLs, seem to play a particularly important role in the regulation of inflammatory responses. A reduced level of plasmalogens is observed in microglia following treatment with inflammatory stimuli like LPS and IL-1β [20]. This is due to the downregulation of *GNPAT* expression by NF-κB-mediated *c-myc* expression and recruitment onto the *GNPAT* promoter [20,21]. sh-RNA-mediated *GNPAT* knockdown has been shown to induce Toll-like receptor 4 (TLR4) endocytosis and proinflammatory cytokine (1L-1β) expression, suggesting that the decreased synthesis of plasmalogens promotes inflammatory responses [20,21,22]. Conversely, plasmalogen treatment can reduce microglial activation in the brains of aged [23] and LPS-treated mice [24]. The altered responses included increased microglial branching and reduced expression of proinflammatory cytokines including IL-1β, IL-6, and TNF-α in the aged brain [23]. Plasmalogen also reduced the activation of multiple inflammatory signaling pathways, including PKCδ activation and NF-κB expression in the brains of LPS-treated mice and transgenic AD (Alzheimer’s disease)-mouse models [21,25], and inhibited p38-MAPK and JNK pathways and nitric oxide (NO) production in LPS-treated microglial cells [26]. Additionally, plasmalogens have been shown to inhibit LPS-induced TLR4 endocytosis [22], possibly due to changes in membrane fluidity [10,27].

Interestingly, *Gnpat*-knockout mice, which are deficient in the peroxisomal ether-PL synthesizing enzymes, do not show substantial neuroinflammation. These mice have been reported to have decreased maturation and a lower number of iNKT cells [13,28] and suffer from demyelination and axonal damage. However, they did not show any noticeable signs of microglial activation or proliferation [29]. This study was carried out using young mice (3 weeks to 5 months old). This suggests that Gnpat deficiency may not affect microglia in young mice. Alternatively, since these mice lack Gnpat during development, microglia in these mice might be developmentally altered and adapted to function in the absence of ether-PLs through a compensatory mechanism. It is also unknown how microglia in these mice respond to the inflammatory stimuli or during aging.

### 2.3. Peroxisomal Redox Metabolism

Peroxisomes contain both ROS (reactive oxygen species)-generating and ROS-degrading enzymes and play an important role in maintaining cellular redox balance [1,2,3]. Many peroxisomal metabolic reactions catalyzed by enzymes such as ACOX1, urate oxidase, D-amino oxidase, polyamine oxidase, and xanthine oxidase generate hydrogen peroxide as a byproduct, which is then decomposed by the peroxisome-resident antioxidant enzyme, catalase [1,2,3] (Figure 1). Peroxisomes also contain other antioxidant enzymes such as superoxide dismutase1, peroxiredoxin 5, and glutathione S-transferase kappa 1 [1,2,3]. When the balance between the ROS-generating and antioxidant enzymes is disrupted, the peroxisomal ROS level increases, leading to cellular oxidative stress.

Neuroinflammation is frequently associated with oxidative stress [6,30,31,32,33]. ROS are generated during inflammation and can trigger inflammatory response in immune cells. ROS-mediated signaling pathways are implicated in microglia and astrocyte activation [31], promoting the expression of proinflammatory cytokines and chemokines. Additionally, activated microglia can act as a major source of extracellular ROS, which can be harmful for neurons [31].

In activated microglia or macrophages, phagosome-associated nicotinamide adenine dinucleotide phosphate (NADPH) oxidases (NOX) generate ROS such as hydrogen peroxide, which activates NF-κB and induces the tyrosine phosphorylation of IκB and IKK activation, causing the expression of proinflammatory cytokines [6,30,31,33,34]. It has been reported that peroxisomes localize near the phagosomes in peritoneal macrophages during phagocytosis [13,34] and discharge catalase to control the oxidative burst within phagosomes [13]. This is extremely important for phagolysosome processing. Macrophages lacking functional peroxisomes show elevated level of hydrogen peroxide that impair phagosomal maturation [13,35]. Catalase overexpression in those macrophages attenuated defects in phagocytosis, suggesting that peroxisomal ROS metabolism is essential to phagosome development and maturation in immune cells [35].

Peroxisomes also regulate adaptive immunity, including T-cell proliferation. Mice lacking peroxisomal biogenesis factor Pex5, required for the delivery of peroxisomal proteins to the pre-peroxisomal structures, have been shown to have a reduced proliferation of stimulated CD4+ helper T cells and CD8+ cytotoxic T cells due to excessive ROS production [13]. Interestingly, Pex5 inactivation, specifically in oligodendrocytes, causes neuroinflammation and triggers the infiltration of CD8+ T cells expressing elevated levels of macrophage inflammatory protein (MIP-1a), interferon gamma, and monocyte chemotactic proteins in the mouse brain [36]. Pex5 deficiency in neural cells also causes neuroinflammation and oxidative stress. This suggests that the perturbation of cellular redox balance due to peroxisomal impairment or deficiency may trigger inflammatory response and monocyte infiltration in the central nervous system.

## 3. Peroxisomes in Innate Immune Signaling during Viral Infection

Peroxisomes serve as the signaling hub for antiviral immune response [37,38,39]. Mitochondrial antiviral signaling (MAVS) protein, an important component of innate immunity that localizes to mitochondria and mitochondria-associated membranes on the ER, has also been detected localized on the peroxisomal membrane [40]. Upon viral infection, retinoic acid inducible gene-I (RIG-1), a cytosolic pattern recognition receptor (PRR), recognizes viral RNA and interacts with MAVS and activates it [37,38,39]. Activated peroxisomal MAVS then triggers cascade of antiviral signaling and induces the expression of type-III interferon (IFN-III), a class of interferon involved in antiviral immune response [38,41]. On the other hand, MAVS activation on mitochondrial membranes mainly induces IFN-I expression [38]. Viruses interfere with peroxisomal MAVS or disrupt peroxisomal biogenesis to evade the antiviral response. Hepatitis C virus (HCV) cleaves MAVS on mitochondrial and peroxisomal membranes by its serine protease complex NS3/4A [42]. Zika virus (ZIKV) infection lowers the peroxisomal pool. Its capsid protein forms a stable complex with the peroxisomal biogenesis factor (peroxin): PEX19 [43]. The capsid proteins of other flaviviruses like dengue virus and West Nile virus also interact with PEX19 and disrupt peroxisomal biogenesis [38]. Human immunodeficiency virus (HIV) has also been shown to impair peroxisomal biogenesis by upregulating miRNAs that specifically target peroxisomal biogenesis factors like PEX2, PEX7, PEX11β, and PEX13 [44]. On the other hand, severe acute respiratory syndrome coronavirus 2 (SARS-CoV-2) blocks peroxisomal antiviral response by affecting peroxisomal functions and integrity. Its protein, ORF14, interacts with the peroxisomal membrane protein PEX14 and prevents the import of peroxisomal proteins, causing a reduction in peroxisomes [45]. Viral proteins also translocate to peroxisomes and inhibit their functions. The HIV protein Nef and the rotavirus structural protein VP4 have been shown to localize to the peroxisomes [38,46,47]. They interfere with the peroxisomal lipid metabolism. Viruses also modulate the peroxisomal lipid metabolism to support their proliferation. Human cytomegalovirus promotes peroxisome biogenesis to boost the production of plasmalogens required for their assembly [48]. An increased plasmalogen level has also been detected in the plasma of patients infected with ZIKV, suggesting that ZIKV probably also enhances peroxisomal ether-phospholipid synthesis to favor its proliferation [49]. These clearly indicate that peroxisomes play an important role in regulating antiviral innate immune response and contribute to neuroinflammation during CNS infection.

## 4. Neuroinflammation in Peroxisomal Disorders

Peroxisomal disorders are a group of congenital diseases caused by a mutation in one or more peroxisomal proteins and leading to impairment in peroxisomal function. Peroxisomal disorders are classified into two categories: (1) peroxisomal biogenesis disorders (PBDs) and (2) single-peroxisomal-protein deficiencies. Both types of peroxisomal disorders have been reported to be associated with neuroinflammation (Table 1).

### 4.1. Peroxisomal Biogenesis Disorders (PBDs)

Peroxisomal biogenesis is regulated by concerted functions of different peroxisomal biogenesis factors (peroxins, PEX) that facilitate the import of peroxisomal proteins to pre-peroxisomal or existing peroxisomal structures. PBDs can be caused by mutations in any of 13 different *PEX* genes that encode peroxins, causing disruption in peroxisome assembly or maturation [1,50]. This results in a decrease in overall peroxisomal activity due to the absence or lower abundance of functional peroxisomes. The main types of PBD include Zellweger spectrum disorders (ZSDs) and rhizomelic chondrodysplasia punctata (RCDP) type 1, and ZSDs include Zellweger syndrome (ZS), neonatal adrenoleukodystrophy (NALD), infantile Refsum’s disease (IRD), and Heimler syndrome [1,51]. ZS is the most severe among them, with patients displaying severe hypotonia, vision impairment, seizures, cerebellar ataxia, neuropathy, hepatic dysfunction, and craniofacial dysmorphology [1,50,51]. They die prematurely in early childhood. NALD and IRD represent the intermediate forms of Zellweger spectrum disorder. They are clinically characterized by hypotonia, hepatic dysfunction, ataxia, sensorineural loss, and vision impairment. NALD patients usually die in their teens, while IRD patients may survive until early adulthood [1,50,51]. Heimler syndrome is the mildest form of all ZSDs. Patients with Heimler syndrome do not present any major developmental delay and mainly suffer from sensorineural loss and nail and retinal pigmentation abnormalities [1]. The majority of ZSDs are caused by a mutation in the *PEX1* or *PEX6* genes, which account for 60% and 16% of all ZSDs, respectively [36]. The second type of PBD, RCDP, is caused by mutations in the *PEX7* or *PEX5L* genes that affect the import of peroxisomal proteins, AGPS, phytanoyl CoA 2-hydroxylase, and 3-ketoacyl-CoA thiolase [50]. The clinical features include the proximal shortening of the long bones (rhizomelia), punctate calcification in the epiphyseal cartilage of the knee, elbow, and shoulder, cataract formation, and intellectual disability [1,50,51].

The extent of neuroinflammation has not been studied thoroughly in patients with peroxisomal biogenesis disorders. However, a severe inflammatory response has been reported in a ZS patient with *PEX6* mutation [52]. Studies carried out in model organisms also support neuroinflammation in ZSDs. Kassmann et al. showed increased levels of proinflammatory cytokines (TNF-α, IL-10, and IFN-γ) and chemokines (MIP-1a, MCP-1, MCP-5, and IP-10) in the brains of oligodendrocyte-specific *Pex5*-knockout mice [36]. They also demonstrated the infiltration of B cells and activated CD8+ T cells into brain lesions in these mice. The activation of innate immune response was also reported in neural-specific *Pex5*-knockout mice. Several proinflammatory markers including TNF-α, complement–C1q, Toll-like receptor–TLR2, and chemokine–Cxcl-1 were markedly increased in the brains of these mice. Both *Pex5* and *Pex7* deficiencies have been shown to impair phagosome formation [35]. Both astro- and microgliosis have been reported in *Pex13*-knockout mice [53].

### 4.2. Single-Peroxisomal-Protein Deficiencies

Single-peroxisomal-protein or -enzyme deficiencies lead to impairments in specific peroxisomal functions, including the oxidation of VLCFAs or branched-chain fatty acids or ether-PL synthesis. Peroxisomal β-oxidation disorders include X-adrenoleukodystrophy (X-ALD) caused by a mutation in the gene of the VLCFA importer *ABCD1* (ATP-binding cassette transporter subfamily D member 1); pseudo-neonatal adrenoleukodystrophy (PNALD) caused by acyl-CoA oxidase 1 deficiency; D-bifunctional protein deficiency (DBPD); and a-methyl-acyl-CoA-racemase (AMACR) deficiency [1,54]. X-ALD is the most common peroxisomal disorder. It is a progressive neurodegenerative disease that affects the central nervous system, peripheral nerves, and adrenal glands [1,54,55]. A lack of functional ABCD1 impairs the delivery of VLCFAs into the peroxisomal lumen for β-oxidation. This causes increased levels of VLCFAs in X-ALD patients who develop severe neurological deficits that include visual dysfunction, motor impairment, and seizures [1,54,55]. Since ACOX1 and DBP are the major components of the peroxisomal β-oxidation pathway, VLCFAs also accumulate in ACOX1 and DBP deficiencies. Clinical features of these two diseases resemble those of ZSDs [1]. AMACR deficiency is a rare disorder, with just 15 patients identified so far. Its clinical features include peripheral neuropathy, cataract, relapsing encephalopathy, thalamic lesion, tremor and liver abnormalities [1]. Defects in the peroxisomal α-oxidation of branched fatty acids cause Refsum disease. Patients affected by a defect in peroxisomal ether-PL synthesis show a clinical phenotype similar to RCDP, including developmental delay, proximal shortening, spasticity, cataracts, and premature death [1]. RCDP type 2 and 3 are caused by mutations in the *GNPAT* and *AGPS* genes that encode for two peroxisomal ether-PL synthesizing enzymes, dihydroxyacetone phosphate (DHAP) acyltransferase and alkyl-DHAP synthase, respectively [1,50].

Inflammatory changes have been most widely studied in X-ALD patients, whose pathophysiology shows progressive inflammatory demyelination [1,54,55,56]. Microglia are activated in the brains of X-ALD patients due to the elevated levels of VLCFAs containing lysophosphatidycholine [55,57]. Different proinflammatory cytokines (IL-1β, TNF-α, and IFN-γ) increase in the demyelination plaques of X-ALD brains. Increased levels of proinflammatory chemokines (IL-8, IL-1ra, MCP-1, and MIP-1b) were also detected in the cerebrospinal fluid of CALD (childhood ALD, the most severe form of X-ALD) patients [57,58]. CALD is associated with disruption to the blood–brain barrier (BBB) and the infiltration of peripheral monocytes, predominantly macrophages, into the brain [57]. Macrophages from these patients induce the expression of matrix metalloproteinases (MMP9 and MMP14), as well as the urokinase plasminogen activator surface receptor (PLAUR) in response to VLCFAs [15]. The MMPs degrade the extracellular matrix and might be responsible for BBB disruption. In addition to macrophages, T cells, mostly CD8 cytotoxic T cells (a/b TCR-positive), have been shown to infiltrate near the site of BBB disruption, and B-cell infiltration has been observed in the white matter [57]. The mechanisms behind impaired VLCFAs degradation in X-ALD macrophages predisposing them to proinflammatory responses have also been studied in animal models and patient-derived cells. Recently, the increased production of 25-hydroxycholesterol due to the upregulation of cholesterol 25-hydroxylase in CALD-patient-derived iPSCs has been implicated in NLRP3 inflammasome activation [59]. *Abcd1*^−/−^ mice, the mouse model of X-ALD, do not show any inflammatory demyelination [60]. However, mild neuroinflammatory changes, including elevated levels of the microglial markers IBA-1 and CD68, have been reported in the spinal cords of 15-month-old *Abcd1*^−/−^ mice [61]. Increased levels of C1q and TREM2 have also been reported in the spinal cords of these mice.

In addition to X-ALD, inflammatory changes have been observed in other diseases with peroxisomal β-oxidation deficiency. Verheijden et al. demonstrated extensive microgliosis in several brain regions, including the medulla oblongata, cerebral cortex, corpus callosum, hippocampus, midbrain, and thalamus, in multifunction-protein-deficient (*Mfp2*^−/−^) mice [62]. They detected elevated levels of proinflammatory markers including TNF-α, IL-1β, IL-6, C1q, and TLR2. Neural-specific MFP2 deficiency (*Nestin- Mfp2*^−/−^) also triggers microgliosis but to a lesser extent than in *Mfp2*^−/−^ mice. This is most likely due to the normal MFP2 function in the microglia of *Nestin- Mfp2*^−/−^ mice, unlike *Mfp2*^−/−^ mice in which MFP2 is absent from all cells including microglia. ACOX1 deficiency may also trigger inflammatory response. The activation of the IL-1 inflammatory pathway in NALD patient (with mutant *ACOX1*) fibroblasts has been reported [63]. *Acox1*^−/−^ mice showed steatohepatitis; however, neuroimmune changes have not been described in these mice [64].

**Table 1:** Neuroinflammatory response in peroxisomal disorders.

Peroxisomal Disorder	Gene Affected	Inflammatory Response	Species	Ref
ZSD	*PEX6*	Severe inflammatory response	Human	[52]
	*Pex5*	Increased expression of proinflammatory cytokines and chemokines Infiltration of B and CD8+ T cells in the brain	Mouse	[36]
	*Pex7*	Defect in phagocytosis	Mouse	[35]
	*Pex13*	Astrogliosis and microgliosis	Mouse	[53]
X-ALD	*ABCD1*	Progressive inflammatory demyelination Microglial activation Proinflammatory cytokine and chemokine expression CD8+ T-cell and B-cell infiltration in the brain	Human	[54,55,56,57,58]
	*Abcd1*	Microgliosis and increased levels of c1q and TREM2 in the spinal cord	Mouse	[61]
RCDP	*GNPAT*	Gliosis	Human	[65]
	*Gnpat*	Astrogliosis	Mouse	[29]

ZSD, Zellweger syndrome disorder; X-ALD, X-adrenoleukodystrophy; RCDP, rhizomelic chondrodysplasia punctata.

Much less is known about inflammatory changes in diseases with altered peroxisomal ether lipid synthesis. RCDP, which is due to ether-PL deficiency, is associated with gliosis. An increased level of myo-inositol, a marker for gliosis, was detected in an RCDP patients’ brain by MR-spectroscopy [65]. Mild astrogliosis has been reported in the brain white matter of ether-lipid-deficient mice (*Gnpat*^−/−^ mice) [29]. However, microgliosis and proinflammatory cytokine expression is less pronounced in these mice [29].

## 5. Perspective

The immunometabolic role of peroxisomes has been established in many studies over the last couple of decades [13,34]. Their role in neuroinflammation has been investigated in patients with genetic peroxisomal disorders, in vivo animal models, and in vitro models. Together, these data overwhelmingly suggests that normal peroxisomal function is essential in restricting neuroinflammation. While congenital peroxisomal disorders are relatively rare, it has been demonstrated that peroxisomal function declines during aging and is further impaired in neurodegenerative diseases including Alzheimer’s disease and Parkinson’s disease. Elevated levels of VLCFAs and decreased levels of plasmalogen have been detected in the postmortem brains of human Alzheimer’s patients [3,9,66]. A reduced level of plasmalogen has also been observed in the postmortem frontal cortex of human PD patients [3,66]. This suggests that peroxisomal function is impaired in age-associated neurodegenerative diseases. This also suggests that determining blood VLCFAs and plasmalogens levels may help in detecting neurodegenerative diseases early. Brain aging and neurodegeneration are both associated with progressive neuroinflammation [67], suggesting the possibility that peroxisomal dysfunction might also be a contributing factor to neuroinflammation in these diseases. Thus, increasing peroxisomal function during aging and in neurodegenerative diseases might be beneficial in restricting neuroinflammation. This can be achieved by increasing functional peroxisomes by inducing their biogenesis, which is regulated by peroxins. Thus, developing suitable treatment strategies to enhance peroxin expression to promote peroxisomal biogenesis might be useful in attenuating inflammatory response in the CNS in pathological conditions. Future studies aiming to elucidate the role of peroxisomes or peroxisomal metabolites in neuroinflammation in neurodegenerative diseases and during aging may also open up new therapeutic avenues for age-associated neurodegenerative diseases.

## References

[1] Wanders R.J.A., Baes M., Ribeiro D., Ferdinandusse S., Waterham H.R. (2023). The physiological functions of human peroxisomes. Physiol. Rev..

[2] Fourcade S., Ferrer I., Pujol A. (2015). Oxidative stress, mitochondrial and proteostasis malfunction in adrenoleukodystrophy: A paradigm for axonal degeneration. Free Radic. Biol. Med..

[3] Islinger M., Voelkl A., Fahimi H.D., Schrader M. (2018). The peroxisome: An update on mysteries 2.0. Histochem. Cell Biol..

[4] De Duve C., Baudhuin P. (1966). Peroxisomes (microbodies and related particles). Physiol. Rev..

[5] de Araújo Boleti A.P., de Oliveira Flores T.M., Moreno S.E., Dos Anjos L., Mortari M.R., Migliolo L. (2020). Neuroinflammation: An overview of neurodegenerative and metabolic diseases and of biotechnological studies. Neurochem. Int..

[6] Simpson D.S.A., Oliver P.L. (2020). ROS Generation in Microglia: Understanding Oxidative Stress and Inflammation in Neurodegenerative Disease. Antioxidants.

[7] Devanney N.A., Stewart A.N., Gensel J.C. (2020). Microglia and macrophage metabolism in CNS injury and disease: The role of immunometabolism in neurodegeneration and neurotrauma. Exp. Neurol..

[8] Leng F., Edison P. (2021). Neuroinflammation and microglial activation in Alzheimer disease: Where do we go from here?. Nat. Rev. Neurol..

[9] Trompier D., Vejux A., Zarrouk A., Gondcaille C., Geillon F., Nury T., Savary S., Lizard G. (2014). Brain peroxisomes. Biochimie.

[10] Dean J.M., Lodhi I.J. (2018). Structural and functional roles of ether lipids. Protein Cell.

[11] He A., Dean J.M., Lodhi I.J. (2021). Peroxisomes as cellular adaptors to metabolic and environmental stress. Trends Cell Biol..

[12] Griffin E.N., Ackerman S.L. (2020). Lipid Metabolism and Axon Degeneration: An ACOX1 Balancing Act. Neuron.

[13] Di Cara F., Savary S., Kovacs W.J., Kim P., Rachubinski R.A. (2023). The peroxisome: An up-and-coming organelle in immunometabolism. Trends Cell Biol..

[14] Nath A.S., Parsons B.D., Makdissi S., Chilvers R.L., Mu Y., Weaver C.M., Euodia I., Fitze K.A., Long J., Scur M. (2022). Modulation of the cell membrane lipid milieu by peroxisomal beta-oxidation induces Rho1 signaling to trigger inflammatory responses. Cell Rep..

[15] Zierfuss B., Buda A., Villoria-González A., Logist M., Fabjan J., Parzer P., Battin C., Vandersteene S., Dijkstra I.M.E., Waidhofer-Söllner P. (2022). Saturated very long-chain fatty acids regulate macrophage plasticity and invasiveness. J. Neuroinflamm..

[16] Vijayan V., Srinu T., Karnati S., Garikapati V., Linke M., Kamalyan L., Mali S.R., Sudan K., Kollas A., Schmid T. (2017). A New Immunomodulatory Role for Peroxisomes in Macrophages Activated by the TLR4 Ligand Lipopolysaccharide. J. Immunol..

[17] Chung H.-L., Ye Q., Park Y.-J., Zuo Z., Mok J.-W., Kanca O., Tattikota S.G., Lu S., Perrimon N., Lee H.K. (2023). Very-long-chain fatty acids induce glial-derived sphingosine-1-phosphate synthesis, secretion, and neuroinflammation. Cell Metab..

[18] Radzikowska U., Rinaldi A.O., Çelebi Z.C., Karaguzel D., Wojcik M., Cypryk K., Akdis M., Akdis C.A., Sokolowska M. (2019). The Influence of Dietary Fatty Acids on Immune Responses. Nutrients.

[19] Dorninger F., Gundacker A., Zeitler G., Pollak D.D., Berger J. (2019). Ether Lipid Deficiency in Mice Produces a Complex Behavioral Phenotype Mimicking Aspects of Human Psychiatric Disorders. Int. J. Mol. Sci..

[20] Hossain M.S., Abe Y., Ali F., Youssef M., Honsho M., Fujiki Y., Katafuchi T. (2017). Reduction of Ether-Type Glycerophospholipids, Plasmalogens, by NF-kappaB Signal Leading to Microglial Activation. J. Neurosci..

[21] Hossain S., Mawatari S., Fujino T. (2023). Plasmalogens inhibit neuroinflammation and promote cognitive function. Brain Res. Bull..

[22] Ali F., Hossain S., Sejimo S., Akashi K. (2019). Plasmalogens Inhibit Endocytosis of Toll-like Receptor 4 to Attenuate the Inflammatory Signal in Microglial Cells. Mol. Neurobiol..

[23] Gu J., Chen L., Sun R., Wang J.-L., Wang J., Lin Y., Lei S., Zhang Y., Lv D., Jiang F. (2022). Plasmalogens Eliminate Aging-Associated Synaptic Defects and Microglia-Mediated Neuroinflammation in Mice. Front. Mol. Biosci..

[24] Hossain M.S., Tajima A., Kotoura S., Katafuchi T. (2018). Oral ingestion of plasmalogens can attenuate the LPS-induced memory loss and microglial activation. Biochem. Biophys. Res. Commun..

[25] Sejimo S., Hossain M.S., Akashi K. (2018). Scallop-derived plasmalogens attenuate the activation of PKCdelta associated with the brain inflammation. Biochem. Biophys. Res. Commun..

[26] Youssef M., Ibrahim A., Akashi K., Hossain M.S. (2019). PUFA-Plasmalogens Attenuate the LPS-Induced Nitric Oxide Production by Inhibiting the NF-kB, p38 MAPK and JNK Pathways in Microglial Cells. Neuroscience.

[27] Bozelli J.C., Azher S., Epand R.M. (2021). Plasmalogens and Chronic Inflammatory Diseases. Front. Physiol..

[28] Facciotti F., Ramanjaneyulu G.S., Lepore M., Sansano S., Cavallari M., Kistowska M., Forss-Petter S., Ni G., Colone A., Singhal A. (2012). Peroxisome-derived lipids are self antigens that stimulate invariant natural killer T cells in the thymus. Nat. Immunol..

[29] Bottelbergs A., Verheijden S., Van Veldhoven P.P., Just W., Devos R., Baes M. (2012). Peroxisome deficiency but not the defect in ether lipid synthesis causes activation of the innate immune system and axonal loss in the central nervous system. J. Neuroinflamm..

[30] Andrés C.M.C., de la Lastra J.M.P., Juan C.A., Plou F.J., Pérez-Lebeña E. (2022). The Role of Reactive Species on Innate Immunity. Vaccines.

[31] Fan H., Bai Q., Yang Y., Shi X., Du G., Yan J., Shi J., Wang D. (2023). The key roles of reactive oxygen species in microglial inflammatory activation: Regulation by endogenous antioxidant system and exogenous sulfur-containing compounds. Eur. J. Pharmacol..

[32] Hsieh H.-L., Yang C.-M. (2013). Role of redox signaling in neuroinflammation and neurodegenerative diseases. BioMed Res. Int..

[33] Solleiro-Villavicencio H., Rivas-Arancibia S. (2018). Effect of Chronic Oxidative Stress on Neuroinflammatory Response Mediated by CD4(+)T Cells in Neurodegenerative Diseases. Front. Cell. Neurosci..

[34] Di Cara F., Andreoletti P., Trompier D., Vejux A., Bülow M.H., Sellin J., Lizard G., Cherkaoui-Malki M., Savary S. (2019). Peroxisomes in Immune Response and Inflammation. Int. J. Mol. Sci..

[35] Di Cara F., Sheshachalam A., Braverman N.E., Rachubinski R.A., Simmonds A.J. (2017). Peroxisome-Mediated Metabolism Is Required for Immune Response to Microbial Infection. Immunity.

[36] Kassmann C.M., Lappe-Siefke C., Baes M., Brügger B., Mildner A., Werner H.B., Natt O., Michaelis T., Prinz M., Frahm J. (2007). Axonal loss and neuroinflammation caused by peroxisome-deficient oligodendrocytes. Nat. Genet..

[37] Cook K.C., Moreno J.A., Beltran P.M.J., Cristea I.M. (2019). Peroxisome Plasticity at the Virus-Host Interface. Trends Microbiol..

[38] Ferreira A.R., Marques M., Ramos B., Kagan J.C., Ribeiro D. (2022). Emerging roles of peroxisomes in viral infections. Trends Cell Biol..

[39] Ferreira A.R., Marques M., Ribeiro D. (2019). Peroxisomes and Innate Immunity: Antiviral Response and Beyond. Int. J. Mol. Sci..

[40] Dixit E., Boulant S., Zhang Y., Lee A.S., Odendall C., Shum B., Hacohen N., Chen Z.J., Whelan S.P., Fransen M. (2010). Peroxisomes are signaling platforms for antiviral innate immunity. Cell.

[41] Odendall C., Dixit E., Stavru F., Bierne H., Franz K.M., Durbin A.F., Boulant S., Gehrke L., Cossart P., Kagan J.C. (2014). Diverse intracellular pathogens activate type III interferon expression from peroxisomes. Nat. Immunol..

[42] Bender S., Reuter A., Eberle F., Einhorn E., Binder M., Bartenschlager R. (2015). Activation of Type I and III Interferon Response by Mitochondrial and Peroxisomal MAVS and Inhibition by Hepatitis C Virus. PLoS Pathog..

[43] Wong C.P., Xu Z., Hou S., Limonta D., Kumar A., Power C., Hobman T.C. (2019). Interplay between Zika Virus and Peroxisomes during Infection. Cells.

[44] Xu Z., Asahchop E.L., Branton W.G., Gelman B.B., Power C., Hobman T.C. (2017). MicroRNAs upregulated during HIV infection target peroxisome biogenesis factors: Implications for virus biology, disease mechanisms and neuropathology. PLoS Pathog..

[45] Knoblach B., Ishida R., Hobman T.C., Rachubinski R.A. (2021). Peroxisomes exhibit compromised structure and matrix protein content in SARS-CoV-2-infected cells. Mol. Biol. Cell.

[46] Cohen G.B., Rangan V.S., Chen B.K., Smith S., Baltimore D. (2000). The human thioesterase II protein binds to a site on HIV-1 Nef critical for CD4 down-regulation. J. Biol. Chem..

[47] Mohan K.V., Som I., Atreya C.D. (2002). Identification of a type 1 peroxisomal targeting signal in a viral protein and demonstration of its targeting to the organelle. J. Virol..

[48] Beltran P.M.J., Cook K.C., Hashimoto Y., Galitzine C., Murray L.A., Vitek O., Cristea I.M. (2018). Infection-Induced Peroxisome Biogenesis Is a Metabolic Strategy for Herpesvirus Replication. Cell Host Microbe.

[49] Queiroz A., Pinto I.F.D., Lima M., Giovanetti M., de Jesus J.G., Xavier J., Barreto F.K., Canuto G.A.B., Amaral H.R.D., de Filippis A.M.B. (2019). Lipidomic Analysis Reveals Serum Alteration of Plasmalogens in Patients Infected With ZIKA Virus. Front. Microbiol..

[50] De Munter S., Verheijden S., Régal L., Baes M. (2015). Peroxisomal Disorders: A Review on Cerebellar Pathologies. Brain Pathol..

[51] Wanders R., Waterham H. (2005). Peroxisomal disorders I: Biochemistry and genetics of peroxisome biogenesis disorders. Clin. Genet..

[52] Lucaccioni L., Righi B., Cingolani G.M., Lugli L., Della Casa E., Torcetta F., Iughetti L., Berardi A. (2020). Overwhelming sepsis in a neonate affected by Zellweger syndrome due to a compound heterozygosis in PEX 6 gene: A case report. BMC Med. Genet..

[53] Müller C.C., Nguyen T.H., Ahlemeyer B., Meshram M., Santrampurwala N., Cao S., Sharp P., Fietz P.B., Baumgart-Vogt E., Crane D.I. (2011). PEX13 deficiency in mouse brain as a model of Zellweger syndrome: Abnormal cerebellum formation, reactive gliosis and oxidative stress. Dis. Models Mech..

[54] Wanders R.J., Waterham H.R. (2006). Peroxisomal disorders: The single peroxisomal enzyme deficiencies. Biochim. Biophys. Acta.

[55] Turk B.R., Theda C., Fatemi A., Moser A.B. (2020). X-linked adrenoleukodystrophy: Pathology, pathophysiology, diagnostic testing, newborn screening and therapies. Int. J. Dev. Neurosci..

[56] Weinhofer I., Rommer P., Gleiss A., Ponleitner M., Zierfuss B., Waidhofer-Söllner P., Fourcade S., Grabmeier-Pfistershammer K., Reinert M.-C., Göpfert J. (2023). Biomarker-based risk prediction for the onset of neuroinflammation in X-linked adrenoleukodystrophy. EBioMedicine.

[57] Berger J., Forss-Petter S., Eichler F. (2014). Pathophysiology of X-linked adrenoleukodystrophy. Biochimie.

[58] Yu J., Chen T., Guo X., Zafar M.I., Li H., Wang Z., Zheng J. (2022). The Role of Oxidative Stress and Inflammation in X-Link Adrenoleukodystrophy. Front. Nutr..

[59] Jang J., Park S., Hur H.J., Cho H.-J., Hwang I., Kang Y.P., Im I., Lee H., Lee E., Yang W. (2016). 25-hydroxycholesterol contributes to cerebral inflammation of X-linked adrenoleukodystrophy through activation of the NLRP3 inflammasome. Nat. Commun..

[60] Rutherford H.A., Hamilton N. (2019). Animal models of leukodystrophy: A new perspective for the development of therapies. FEBS J..

[61] Manor J., Chung H., Bhagwat P.K., Wangler M.F. (2021). ABCD1 and X-linked adrenoleukodystrophy: A disease with a markedly variable phenotype showing conserved neurobiology in animal models. J. Neurosci. Res..

[62] Verheijden S., Bottelbergs A., Krysko O., Krysko D.V., Beckers L., De Munter S., Van Veldhoven P.P., Wyns S., Kulik W., Nave K.-A. (2013). Peroxisomal multifunctional protein-2 deficiency causes neuroinflammation and degeneration of Purkinje cells independent of very long chain fatty acid accumulation. Neurobiol. Dis..

[63] El Hajj H.I., Vluggens A., Andreoletti P., Ragot K., Mandard S., Kersten S., Waterham H.R., Lizard G., Wanders R.J., Reddy J.K. (2012). The inflammatory response in acyl-CoA oxidase 1 deficiency (pseudoneonatal adrenoleukodystrophy). Endocrinology.

[64] Huang J., Viswakarma N., Yu S., Jia Y., Bai L., Vluggens A., Cherkaoui-Malki M., Khan M., Singh I., Yang G. (2011). Progressive endoplasmic reticulum stress contributes to hepatocarcinogenesis in fatty acyl-CoA oxidase 1-deficient mice. Am. J. Pathol..

[65] Viola A., Confort-Gouny S., Ranjeva J.-P., Chabrol B., Raybaud C., Vintila F., Cozzone P.J. (2002). MR imaging and MR spectroscopy in rhizomelic chondrodysplasia punctata. AJNR Am. J. Neuroradiol..

[66] Cipolla C.M., Lodhi I.J. (2017). Peroxisomal Dysfunction in Age-Related Diseases. Trends Endocrinol. Metab..

[67] Andronie-Cioara F.L., Ardelean A.I., Nistor-Cseppento C.D., Jurcau A., Jurcau M.C., Pascalau N., Marcu F. (2023). Molecular Mechanisms of Neuroinflammation in Aging and Alzheimer’s Disease Progression. Int. J. Mol. Sci..

